# Evaluating breast ultrasonography as a complementary diagnostic method in girls with central precocious puberty

**DOI:** 10.1007/s00247-024-05934-4

**Published:** 2024-05-08

**Authors:** Erhan Bıyıklı, Didem Helvacıoğlu, Onur Buğdaycı, Buşra Gürpınar Tosun, Serap Turan, Tülay Güran, Abdullah Bereket

**Affiliations:** 1 Department of Radiology, Marmara University, School of Medicine, Istanbul, Turkey; 2 Division of Pediatric Endocrinology, Department of Pediatrics, Marmara University, School of Medicine, Maltepe 34854 Istanbul, Turkey

**Keywords:** Breast, Central precocious puberty, Elastography, Lipomastia, Luteinizing hormone, Pediatric, Thelarche, Ultrasound

## Abstract

**Background:**

Assessment of breast development by physical examination can be difficult in the early stages and in overweight girls.

**Objective:**

To investigate ultrasonography (US) for evaluation of early breast development.

**Materials and methods:**

In a prospective study, 125 girls (age 7.1 ± 1.5 years) with breast development before 8 years underwent US breast staging, breast volume, and elastography, in addition to clinical/hormonal evaluation for precocious puberty. Accuracy of US for determining breast development and predicting progression to central precocious puberty was investigated.

**Results:**

Physical examination revealed glandular breast enlargement in 100 and predominantly lipomastia in 25. Breast US in the former confirmed glandular breast development in 92 (group 1, physical examination and US positive), but not in 8 (group 2, physical examination positive, US negative). Comparison of the two groups demonstrated lower Tanner and US staging, bone age/chronological age, basal luteinizing hormone (LH), breast volume, and uterine volume in group 2. In the 25 lipomastia patients, US demonstrated no breast tissue in 19 (group 3, physical examination and US negative), but US stage ≥ II in 6 (group 4, physical examination negative, US positive) without differences in clinical parameters. After follow-up of 19.8 ± 4.2 months, 46/125 subjects were diagnosed with precocious puberty. US stage, total breast volume, and shear-wave speeds were significantly higher in these 46 patients. Multivariate analyses demonstrated breast volume > 3.4 cc had odds ratio of 11.0, sensitivity of 62%, and specificity of 89, in predicting progression to precocious puberty, being second only to stimulated LH for all variables.

**Conclusion:**

Breast US is a useful predictive tool for diagnosis of precocious puberty in girls. Higher US stages and higher breast volume on US increased the likelihood of eventual diagnosis of precocious puberty.

**Graphical Abstract:**

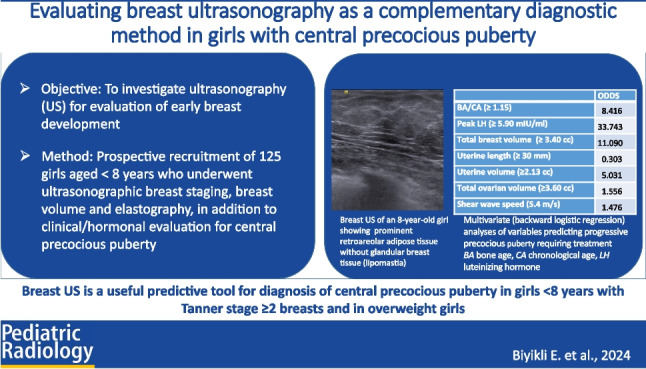

## Introduction

Onset of breast development in girls before 8 years of age is considered the first sign of precocious puberty. It has been demonstrated that overweight/obesity is associated with earlier breast development, as well as earlier menarche in girls [1, 2]. However, it is often difficult to obtain a correct assessment of the breast glandular tissue in overweight/obese girls. Distinguishing mammary tissue from the surrounding loose fat and connective tissue is not always easy and can lead to an inaccurate assessment, possibly resulting in misdiagnosis of breast enlargement in a girl with lipomastia or conversely true breast enlargement may be masked by fat tissue in an overweight/obese girl, delaying the diagnosis of precocious puberty, especially in the early stages of puberty. Furthermore, breast development is not always a sign of progressive precocious puberty and may remain as premature thelarche [3]. It is important to differentiate precocious puberty from premature thelarche since central nervous system (CNS) imaging and treatment with gonadotropin-releasing hormone (GnRH) analogues are required in the former, to exclude an organic pathology and to prevent unwanted effects of early sexual development, respectively.

Tanner staging by physical examination (PE), height velocity, bone age advancement, progression of pubertal signs, GnRH-stimulated gonadotropin levels, and ultrasonographic (US) evaluation of the uterus and ovaries are the main diagnostic tools in the evaluation of a girl with early breast development [4]. However, persistent diagnostic difficulties in the early stages of puberty and in overweight girls led to the search for additional tools in the diagnosis of precocious puberty.

Recently, studies using US evaluation of the breasts have been published. Garcia et al. [5]. reported US characteristics of breasts in children, correlating with Tanner stages 1 through 5.

It has been proposed that US staging of the breasts could be more objective, simple, and reproducible than conventional Tanner staging, but data is conflicting in this regard [6–10]. Furthermore, despite being a promising modality, usefulness of ultrasonographic evaluation of the breast in differentiating progressive versus non-progressive precocious puberty in girls has not yet been adequately studied.

In addition to staging of breast maturation, US enables quantitative assessment of glandular breast volume and elastography. Shear-wave elastography is mainly used to characterize breast lesions and increase the specificity of US in adults, but it has not been used in children to assess the maturation of breast tissue during puberty [11]. Another new US method is uterine artery Doppler analysis which has been reported to assist the diagnosis of central precocious puberty [12].

The aim of the present study is to determine the utility of US evaluation of breast tissue, using morphological characteristics described by Garcia et al. [5] as well as breast volume and elastography in girls with early breast enlargement and to investigate the correlation between breast US parameters with Tanner staging and hormonal and clinical diagnostic criteria for precocious puberty.

## Materials and methods

In this prospective study, girls who were referred to our department for appearance of breast enlargement before the age of 8 years were recruited consecutively between May 2019 and January 2022.

**Fig. 1:**
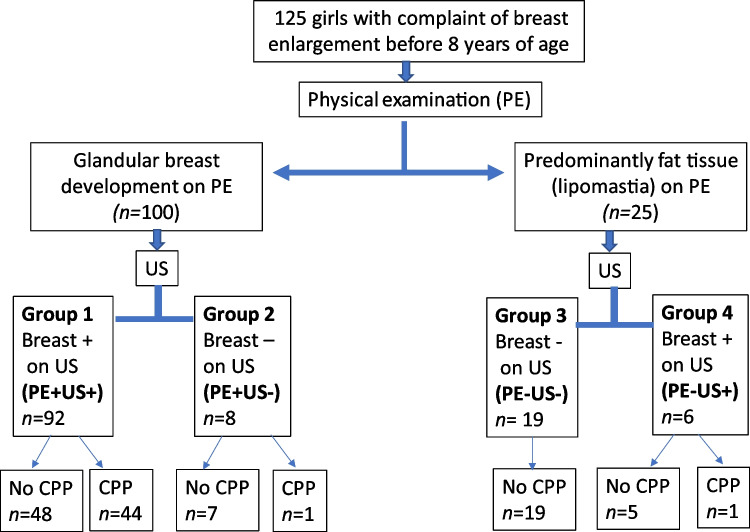
Study outline. *CPP *central precocious puberty, *PE +* physical examination confirmed palpable glandular breast development on physical examination, *PE*- physical examination did not confirm palpable glandular breast development on physical examination, *US +* ultrasound evaluation confirmed glandular breast development, *US-* ultrasound evaluation did not confirm glandular breast development

Inclusion criteria were having a complaint of breast development noticed before 8 years of age, determination of glandular or lipoid breast enlargement on physical examination, and giving consent for inclusion in the study. Patients who clearly had palpable glandular breast tissue on physical examination formed one group (*n* = 100); patients who had the appearance of breast enlargement but had softer consistency on palpation hence were judged to have predominantly lipomastia formed the second group (*n* = 25) (Fig. 1). None of the patients had neurological signs or symptoms or known CNS pathology (except for one girl with spina bifida). Diagnostic evaluation was performed in University Hospital Pediatric Endocrinology Clinic by experienced board-certified pediatric endocrinologists (D.H., A.B., T.G., S.T., and B.G.T.), with experience ranging from 5 to 27 years and included measurements of height and weight, body mass index, physical examination with Tanner staging, bone age assessment, measurement of basal serum follicle stimulating hormone (FSH), luteinizing hormone (LH) and estradiol concentrations, and uterine and ovarian measurements by pelvic US. Pubertal development was staged according to the Marshall and Tanner criteria [13]. Palpable breast budding in at least one breast was considered Tanner stage 2 breast development. Both breasts were staged separately in each patient and in case of asymmetric breast development, the value for the more developed breast was taken and used in the calculations. Height and weight were measured using a Harpenden stadiometer (Holtain Ltd. Crosswell, Crymyh Pembs UK). Body mass index was calculated using the standard formula (weight in kg/height in m^2^), and the respective standard deviation scores (SDS) were calculated, based on local reference data [14–16]. Bone age was assessed from radiographs of the left hand, according to the method of Greulich and Pyle [17]. Bone age to chronological age ratio was calculated for each patient. Fasting venous blood samples were obtained from all girls in the morning for FSH, LH, and estradiol measurements using semi-automated chemiluminescence methods. In patients with basal LH < 0.6 mIU/mL, a GnRH stimulation test was performed.

The criteria for the diagnosis of central precocious puberty were (1) progressive breast development starting before 8 years of age, associated with accelerated growth (> 6 cm/year) and skeletal maturation (advanced by at least 1 year) [18]; and (2) a basal LH > 0.6 mIU/mL or a GnRH-stimulated LH of at least 5 mIU/mL [4, 18–24] as accepted previously in the literature and consensus papers. Uterine length > 34 mm and ovarian volume > 2 cubic centimeters (cc) were taken as supportive criteria [4, 25, 26].

Breast US and shear-wave US elastography were performed in the Radiology Department in a supine position using an Acuson S2000 device (Siemens Healthineers, Erlangen, Germany) with a 9L4 linear array probe (4–9 MHz operating range) by a single board-certified radiologist (E.B.) who has 7 years of experience in evaluating pediatric endocrinology patients. The operator was blinded to the clinical information of the patients.

Breast development evident by US examination was graded according to the system proposed by Garcia et al. [5] who described five morphological stages of glandular development associated with conventional Tanner stages of breast development. These are:

Tanner stage 1, US stage I—US scan shows ill-defined hyperechoic retroareolar tissue.

Tanner stage 2, US stage II—US scan shows a hyperechoic retroareolar nodule with a central hypoechoic area that represents mostly simple branched ducts.

Tanner stage 3, US stage III—US scan shows hyperechoic glandular tissue extending away from the retroareolar area and a central spider-shaped hypoechoic retroareolar region.

Tanner stage 4, US stage IV—US scan shows hyperechoic fibroglandular periareolar tissue with a prominent central retroareolar hypoechoic nodule.

**Fig. 2:**
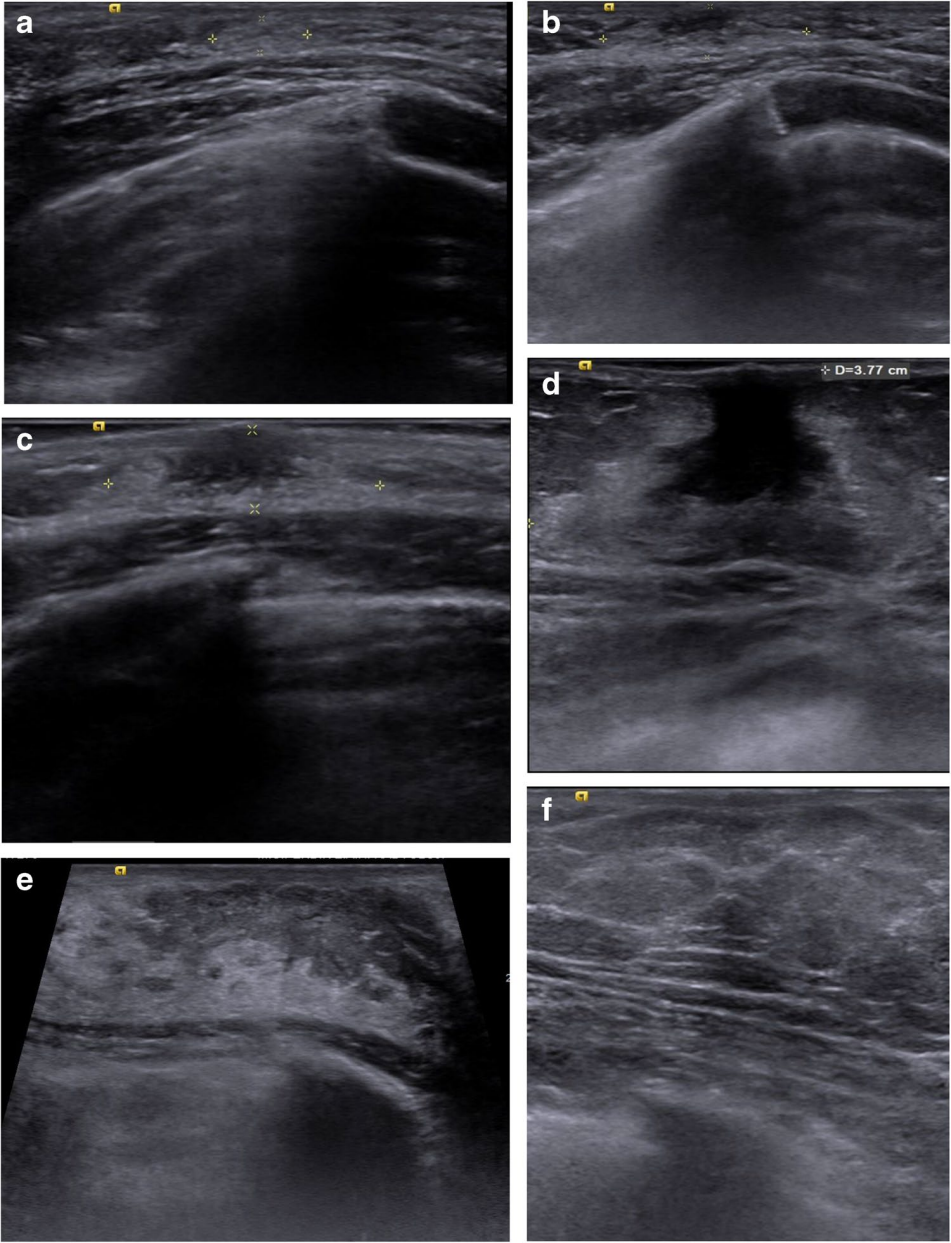
Axial plane grayscale breast ultrasound images show ultrasonographic staging of breast development (**a**–**e**) and lipomastia (**f**). **a** Stage I in a 6-year-old girl with an ill-defined hyperechoic area in the retroareolar zone (*calipers*). **b** Stage II in a 7-year-old girl with a hypoechoic core and hyperechoic nodule limited to the retroareolar area (*calipers*). **c** Stage III in an 8-year-old girl with a spider-shaped hypoechoic nodule within hyperechoic tissue exceeding subareolar area (*calipers*). **d** Stage IV in an 8.5-year-old girl with significant amount of hyperechoic tissue with secondary branching hypoechoic nodule. **e** Stage V in a 12-year-old girl with prominent hyperechoic tissue without central nodule. **f** An 8-year-old girl with prominent retroareolar adipose tissue but without any glandular breast tissue (lipomatous pattern)

Tanner stage 5, US stage V—US scan shows hyperechoic glandular tissue, with increased subcutaneous adipose tissue anteriorly and without the hypoechoic central nodule seen in previous Tanner stages (Fig. 2).

**Fig. 3:**
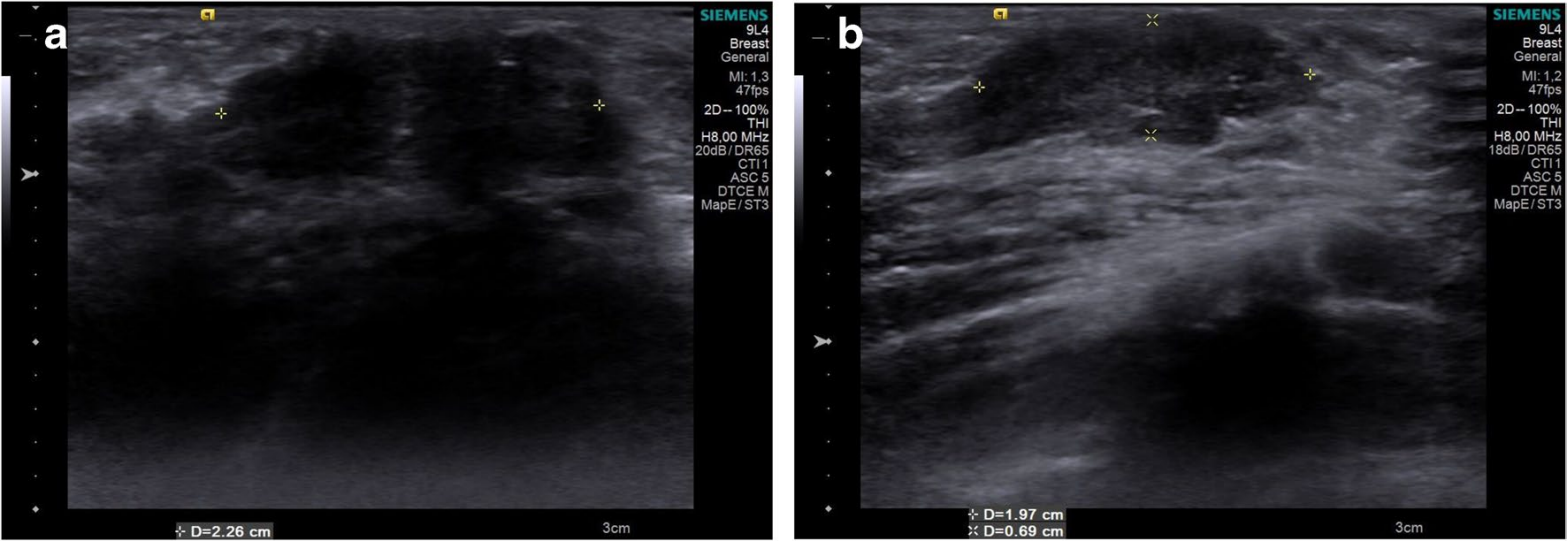
Grayscale ultrasound volume measurements of a stage III breast bud in a 4-year-old girl who was diagnosed with premature thelarche. **a** Longitudinal plane image shows the superoinferior measurement (*calipers*). **b** Axial plane image shows the mediolateral (*calipers* +) and anteroposterior (*calipers* x) measurements. Breast volume was calculated to be 1.6 cc

Breast retroareolar glandular volume was calculated by measuring the glandular area (including the echogenic fibroglandular tissue) in three dimensions using the formula:

Fibroglandular volume = Length × Width × Height × 0.520 (Fig. 3).

**Fig. 4:**
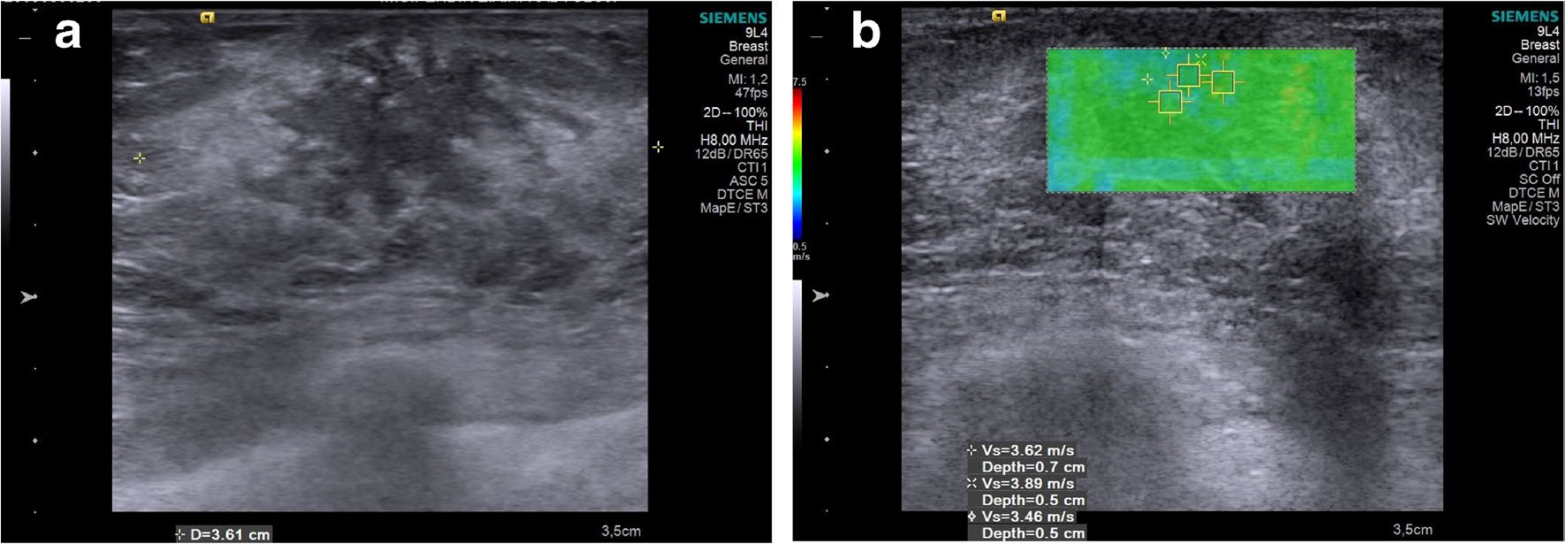
Shear-wave elastography assessment in an 8-year-old girl with a stage III breast bud who was diagnosed with premature thelarche. Grayscale axial ultrasound without (**a**) and with (**b**) shear-wave speed measurements at different locations (*squares*). Mean shear wave velocity was calculated to be 3.66 m/s (**b**)

Shear-wave US elastography measurements were performed on the slice with the largest fibroglandular width, applying as little pressure as possible. A 2 × 2 mm square region of interest (ROI) was placed in three different locations on the slice, based on the color map paying attention to include those parts of the hypoechoic tissue with the highest shear velocities (Fig. 4). The average of these three values was recorded in meters per second.

For breast volume and breast elastography, the sum of the two breasts was used for presentation and analysis.

Clinical evaluation of the rate of progression of pubertal development was performed after a follow-up period of at least 6 months. All girls with rapidly progressive precocious puberty underwent cranial magnetic resonance imaging and treatment with GnRH analog.

Informed consent was obtained from each parent or guardian and the study protocol was approved by the ethical committee of our institution.

Statistical evaluation was performed using Number Cruncher Statistical System2020 Statistical Software (NCSS LLC, Kaysville, UT). Quantitative data are expressed as mean ± standard deviation (SD), or median and range as appropriate. Distribution of the data was evaluated by the Shapiro–Wilk test and Box Plot graphics. Pairwise comparisons were performed using an independent samples test (Student *t* test) when data was normally distributed. The non-parametric Mann–Whitney *U* test was used when the normality assumption of data distribution was absent. Bonferroni’s correction was used where appropriate. Statistical significance was set at *P* < 0.05.

Diagnostic accuracy (sensitivity, specificity, positive predictive value, negative predictive value) and receiver operating characteristic (ROC) curve assessments were used to determine the most appropriate parameters and cut-offs for potential clinical predictors of progressive precocious puberty requiring treatment. Dependent variables included being in either the treatment or no treatment group, whereas breast volume by US, elastography results, peak LH, uterine volume, uterine length, and total ovarian volume were independent variables. The optimal cut-off values were evaluated using the Youden index (*J*) which is defined as *J* = maximum (sensitivity + specificity − 1). Multivariate evaluations were performed using backward logistic regression analyses. Significance of the model was evaluated by the chi-square test. Pearson’s correlation coefficient for continuous variables was used to investigate the associations between the variables.

## Results

In total, 125 girls were included in the study. Physical examination revealed glandular breast enlargement (Tanner stage ≥ 2) in 100 and lipomastia in 25 (Fig. 1). Breast US in the former group confirmed the presence of glandular breast development in 92, designated as group 1 (physical examination and US positive), but not in eight, designated as group 2 (physical examination positive, US negative) (Fig. 1). All girls with Tanner stage ≥ 3 breast development on physical examination were also reported to have glandular breast tissue on US, whereas all group 2 subjects had Tanner stage 2 breasts on physical examination. Median (25–75%) body mass index SDS tended to be higher in group 2 (0.82 [-0.5–1.3]) compared to group 1 (0.44 [-0.2–1.2]) (*P* = NS).

Comparison of the eight group 2 patients with the 92 group 1 patients demonstrated lower Tanner and ultrasonographic staging, bone age/chronological age, US breast volume, uterine length, uterine volume, and ovarian volume in the former. Basal and GnRH-stimulated LH concentrations were also lower in group 2, respectively, but did not reach statistical significance (Table 1).

**Table 1:** Auxological, clinical, and ultrasonographic characteristics of 100 girls with glandular breast tissue on physical examination

	**Group 1** **Physical examination(+)** **US(+)** **(*n *= 92)**	**Group 2** **Physical examination(+)** **US(-)** **(*n *= 8)**	*P*
Chronological age	7.1 ± 1.6	6.2 ± 1.1	0.06
^a^Breast stage, Tanner	2(2–4)	2(2–2)	0.014
^a^Breast stage, ultrasonographic	3 (2–5)	1(1)	< 0.0001
^b^Body mass index SDS	0.44 (-0.2–1.2)	0.82 (-0.5–1.3)	0.65
Bone age	8.2 ± 2.1	5.9 ± 1.8	0.04
Bone age/Chronological age	1.1 ± 0.1	0.9 ± 0.2	0.04
Basal LH (mIU/mL)	0.69 ± 1.2	0.28 ± 0.12	0.09
Peak LH (mIU/mL)	9.5 ± 8.4	4.3 ± 5.6	0.39
Total breast volume (cc)	8.3 ± 8.7	0.45 ± 0.28	< 0.0001
Shear wave speed (m/s)	5.5 ± 1.3	-	NA
Uterine length (mm)	33.1 ± 5.9	26.5 ± 6.1	0.038
Total ovarian volume (cc)	4.2 ± 2.4	2.1 ± 0.7	0.02
Uterine volume (cc)	2.6 ± 1.6	1.0 ± 0.4	0.008
Treatment; *n (%)*	44 (47.8%)	1(12.5%)	NA

Mean ± standard deviation, ^a^median (min–max), ^b^median (25–75%)

*LH* luteinizing hormone, *NA* not applicable, *SDS* standard deviation scores, *US(*+*)* ultrasound also confirmed the presence of glandular breast tissue development, *US(-)* ultrasound did not show glandular breast tissue development

In the 25 lipomastia patients, the median (25–75%) body mass index SDS was 1.77 (1.26–2.1) and significantly higher than found in the 100 patients with palpable breasts 0.47 (-0.22–3.79) on physical examination (*P* < 0.0001).

Breast US in the lipomastia patients demonstrated no glandular breast tissue in 19 (both physical examination and US negative) designated as group 3, but ultrasonographic stage > II in 6 designated as group 4 (physical examination negative, US positive). Besides ultrasonographic scores, the only differences in the features associated with precocious puberty were higher bone age and US breast volumes in group 4 (Table 2). Body mass index SDSs were similar between group 3 and group 4. However, when compared with group 1 patients, median (95% CI) body mass index SDS of group 4 patients was significantly higher ((0.44 [-0.2–1.2] vs 1.54 [1.26–2.13]), *P* < 0.001).

**Table 2:** Auxological, clinical, and ultrasonographic characteristics of 25 girls presenting with early breast development and judged to have lipomastia

	**Group 3** **Physical examination(-)** **US(-)** **(*n *= 19)**	**Group 4** **Physical examination(-)** **US(+)** **(*n *= 6)**	*P*
Chronological age	6.8 ± 1.2	7.4 ± 0.6	0.28
^a^Breast stage, Tanner	1 (0)	1 (0)	NA
^a^Breast stage, ultrasonographic	1 (0)	2.5 (2–3)	< 0.0001
Body mass index SDS	1.6 ± 0.9	1.6 ± 0.4	0.95
Bone age	7.1 ± 1.2	8.6 ± 0.9	0.01
Bone age/Chronological age	1.1 ± 0.1	1.1 ± 0.1	0.11
Basal LH (mIU/mL)	0.30 ± 0.10	0.35 ± 0.15	0.54
Peak LH (mIU/mL)	4.2 ± 2.2	5.2 ± 2.7	0.47
Total breast volume (cc)	0.39 ± 0.2	2.04 ± 1.9	0.02
Shear wave speed (m/s)	-	3.5 ± 1.5	NA
Uterine length (mm)	28.6 ± 5.3	28.5 ± 4.5	0.94
Total ovarian volume (cc)	2.04 ± 1.6	2.8 ± 1.1	0.12
Uterine volume (cc)	1.2 ± 1.0	1.3 ± 0.8	0.52
Treatment; *n* (%)	0	1 (%16.67)	NA

Mean ± standard deviation, ^a^median (min–max), ^b^median (25–75%),

*LH* luteinizing hormone, *NA* not applicable, *SDS* standard deviation scores, *US(*+*)* ultrasound also confirmed the presence of glandular breast tissue development, *US(-)* ultrasound did not show glandular breast tissue development

Measured elastography values ranged from 1.96–8.56 m/s in US-positive patients. After clinical evaluation and follow-up of a mean duration of 19.8 ± 4.2 months, 46/125 (36.8%) were diagnosed with progressive precocious puberty based on the criteria given above and were started on treatment. Forty-four (95.6%) were from group 1, one patient each from group 2 (2.2%), and group 4 (2.2%). In addition to clinical and hormonal parameters, median ultrasonographic stage (III [1–5]) vs II [1–4]), median (25–75%) total breast volume (10.0 [4.6–14.5] vs 2.6 [0.6–6.0]), and shear-wave speed (5.9 ± 1.1 m/s vs 4.9 ± 1.4 m/s) were significantly higher in the group requiring treatment (*n* = 46) compared to those who did not require treatment (*n* = 79) (*P* < 0.01 for all) (Table 3).

**Table 3:** Auxological, clinical, and ultrasonographic characteristics of girls who did (*n*=46) and did not (*n*=79) receive treatment for development of central precocious puberty

	**Treatment (*n *= 46)**	**No treatment (*n *= 79)**	*P*^a^
Chronological age	7.7 ± 0.1	6.7 ± 1.7	**0.0007**
^a^Breast stage, Tanner	3 (1–4)	2 (1–4)	**< 0.0001**
^a^Breast stage, ultrasonographic	3 (1–5)	2 (1–4)	**< 0.0001**
Body mass index SDS	0.59 ± 0.9	0.75 ± 1.2	0.4
Bone age	9.1 ± 1.4	7.2 ± 2.1	**< 0.0001**
Bone age/Chronological age	1.19 ± 0.1	1.0 ± 0.1	**0.001**
Basal LH (mIU/mL)	0.80 ± 0.9	0.34 ± 0.2	**< 0.001**
Peak LH (mIU/mL)	13.2 ± 9.5	4.4 ± 2.2	**< 0.0001**
^b^Total breast volume (cc)	10.0 (4.6–14.5)	2.6(0.6–6.0)	**< 0.0001**
Shear wave speed (m/s)	5.9 ± 1.1	5.0 ± 1.4	**0.0005**
Uterine length (mm)	34.7 ± 5.6	30.0 ± 5.8	**< 0.0001**
Total ovarian volume (cc)	4.6 ± 2.6	3.2 ± 2.0	**0.0006**
Uterine volume (cc)	2.9 ± 1.8	1.8 ± 1.3	**< 0.0001**

Mean ± standard deviation, ^a^Bold represents statistical significance (*P *< 0.05) ^b^median (min–max), ^c^median (25–75%)

*LH* luteinizing hormone, *NA* not applicable, *SDS* standard deviation scores, *US(*+*)* ultrasound also confirmed the presence of glandular breast tissue development, *US(-)* ultrasound did not show glandular breast tissue development

ROC analyses of potential predictors of progressive precocious puberty demonstrated total breast volume > 3.4 cc had a sensitivity of 61.8% and a specificity of 88.6% (*P* < 0.0001). Shear-wave speed > 5.4 m/s had a sensitivity of 64.8% and specificity of 69.7% (*P* < 0.001). For peak LH > 5.9 mIU/mL, these values were 83.3% and 84.4% (*P* < 0.001), and for uterine volume > 2.1 cc these were 68.6% and 81.0%, respectively (*P* < 0.001) (Table 4). When the combination of LH > 5.9 and US breast volume > 3.4 cc was tested, sensitivity (1.00), specificity (0.893), positive predictive value (0.912), and negative predictive value (1.00) increased and outperformed the values obtained by stimulated LH alone.

**Table 4:** Diagnostic accuracy assessments and receiver operating characteristics curve analyses results of clinical and ultrasonographic parameters for predicting progressive precocious puberty requiring treatment

	Diagnostic accuracy assessment					ROC curve		*P*^a^
	Cut-off level	Sensitivity	Specificity	Positive predictive value	Negative predictive value	Area	95% confidence interval	
Peak LH (mIU/mL)	≥ 5.90	83.33	84.44	83.70	84.44	0.883	0.809–0.956	**0.001**
Total breast volume (cc)	≥ 3.40	88.64	61.76	60.00	89.36	0.818	0.737–0,899	**0.001**
Shear-wave speed (m/s)	≥ 5.4	72.09	62.96	60.78	73.91	0.701	0.597–0.805	**0.001**
Uterine volume (cc)	≥ 2.13	68.69	81.00	62.00	85.26	0.739	0.651–0.826	**0.001**
Bone age/chronological age	≥ 1.15	60.87	74.36	58.33	76.32	0.710	0.619–0.800	**0.001**
Uterine length (mm)	≥ 30	86.67	50.63	50.00	86.96	0.719	0.627–0.811	**0.001**
Total ovarian volume (cc)	≥ 3.60	65.22	67.09	53.57	76.81	0.676	0.549–0.772	**0.001**

^a^Bold represents statistical significance (*P *< 0.05)

When we evaluated the effects of bone age/chronological age peak LH, total breast volume, uterine length, uterine volume, total ovarian volume, and shear-wave speed measurements on the diagnosis of progressive precocious puberty with backward logistic regression analysis, the effects of the model on bone age/chronological age (*P* < 0.05), peak LH (*P* < 0.01), and total breast volume (*P* < 0.01) of the model were found to be significant (chi-square = 60.023; *P* = 0.001) and the explanatory coefficient of the model remained significant. The effects of other variables were not found to be significant. The odds ratio of the effect of peak LH cut-off level of 5.90 mIU/mL and above on the diagnosis of precocious puberty was 33.7 (95% CI, 6.7–169.5), the effect of the ultrasonographic total breast volume cut-off of 3.40 and above on the diagnosis of precocious puberty was 11.0 (95% CI, 2.0–60.2), and the effect of bone age/chronological age cut-off of 1.15 and above on the diagnosis of progressive precocious puberty was 8.4 (95% CI, 1.6–44.3) (Table 5).

**Table 5:** Multivariate (backward logistic regression) analyses of variables predicting progressive precocious puberty requiring treatment

	B	*P*^a^	Odds ratio	95% CI for odds	
				Lower	Upper
Bone age/chronological age (≥ 1.15)	2.130	**0.012**	8.416	1.596	44.363
Peak LH (≥ 5.90)	3.519	**0.000**	33.743	6.717	169.510
Total breast volume (≥ 3.40)	2.399	**0.006**	11.009	2.011	60.268
Uterine length (≥ 30)	-1.193	0.368	0.303	0.023	4.069
Uterine volume (≥ 2.13)	1.616	0.188	5.031	0.453	55.815
Total ovarian volume (≥ 3.60)	0.442	0.623	1.556	0.267	9.067
Shear-wave speed (≥ 5.4)	0.389	0.687	1.476	0.222	9.794

^a^Bold represents statistical significance (*P* < 0.05)

Correlations between clinical and US parameters (Table 6) demonstrated that Tanner stage correlated well with US stage (*r* = 0.64, *P* < 0.0001) and total breast volume (*r* = 0.57, *P* < 0.0001). In total, 44/125 (35%) patients had asymmetric breast development. For the total group, correlation of the breast stages between physical examination and US for the left breast was *r* = 0.71 and for the right breast was *r* = 0.68 (*P* < 0.0001 for both). In asymmetric cases, these figures were *r* = 0.63 for the left breast and *r* = 0.56 for the right breast (*P* < 0.001 for both).

**Table 6:** Correlations between clinical and radiological diagnostic parameters

Variable	*r*	US breast stage	Bone age/chronological age	Peak LH	Shear-wave speed	Uterine volume	Total breast volume	Total ovarian volume	Uterine length	Bone age
	***P***									
**Tanner stage**		0.64 < 0.0001	0.24 0.0112	0.41 0.0001	0.38 0.0001	0.38 < 0.0001	0.57 < 0.0001	0.31 0.0003	0.31 0.0007	0.24 0.0066
**US breast stage**			0.24 0.0112	0.41 0.0001	0.37 0.0002	0.42 < 0.0001	0.58 < 0.0001	0.29 0.0014	0.39 < 0.0001	0.34 0.0006
**BA/CA**				0.19 0.0832	0.02 0.8487	0.2 0.0519	0.34 0.0003	0.19 0.1471	0.14 0.1817	0.56 < 0.0001
**Peak LH**					0.36 0.001	0.34 0.0009	0.62 < 0.0001	0.13 0.1721	0.29 0.0048	0.32 0.0032
**Shear-wave speed**						0.33 0.0007	0.27 0.007	0.26 0.0077	0.34 0.0005	0.14 0.1696
**Uterine volume**							0.52 < 0.0001	0.63 < 0.0001	0.74 < 0.0001	0.41 < 0.0001
**Total breast volume**								0.27 0.0003	0.52 < 0.0001	0.47 < 0.0001
**Total ovarian volume**									0.59 < 0.0001	0.35 < 0.0001
**Uterine length**										0.44 < 0.0001
**Bone age**										

*BA* bone age, *CA* chronological age, *LH* luteinizing hormone, *US* ultrasonographic

When subjects were subdivided according to body mass index SDS of < 1 or > 1, correlation between Tanner stage and US breast volume was slightly better in those with body mass index SDS < 1 compared to those with body mass index SDS > 1 (*r* = 0.66 vs *r* = 0.61).

Among all clinical, laboratory, and radiological parameters, US breast volume had the strongest correlation with peak LH (*r* = 0.62, *P* < 0.0001), bone age (*r* = 0.47 *P* < 0.001), and bone age/chronological age (*r* = 0.36, *P* < 0.0001) (Table 6).

## Discussion

We have demonstrated good correlation between morphology of the breasts on US, using the staging described by Garcia et al. [5], and Tanner staging, with US confirming breast development in 92 of 100 girls who had Tanner stage ≥ 2. The likelihood of progression to precocious puberty was 48% in those with breast tissue confirmed with US (US stage ≥ II), whereas this was only 13% in those with breast tissue not confirmed on US (US stage I). All eight girls with US stage I (physical examination positive, US negative) had Tanner stage 2 on physical examination, suggesting that breast development can be missed on US at the beginning of puberty. However, only one girl in this group progressed to precocious puberty, suggesting that when breast enlargement identified by physical examination is not confirmed by US, progressive precocious puberty is unlikely.

We also explored the possibility of missing glandular breast tissue development on physical examination by doing breast US in a separate group of girls who presented with breast development but were judged to have predominantly fat tissue on physical examination (*n* = 25). As expected, these girls had higher body mass index than the first group of patients. US stages were ≥ II in 6/25 (24%), suggesting that breast development can be missed by physical examination in some overweight/obese girls. Nevertheless, only one girl (a 7-and-a-half-year-old, with body mass index SDS of 2.12, who had US breast volume of 9.5 cc) needed treatment during follow-up.

Thus, it appears that at the beginning of breast development, both Tanner staging and US morphological staging have a fair ability to distinguish girls with rapidly progressive central precocious puberty from those with non- or slowly progressive or transient forms. However, combining both staging methods increased the detection power of these tools in isolation, especially in those with Tanner stage 2 breasts with soft consistency and those with higher body mass index. Furthermore, besides US morphological staging of the breasts, measurement of total breast volume by US appeared to be a useful marker for progressive precocious puberty with a cut-off volume of > 3.4 cc having highest odds ratio, sensitivity, and specificity emerging as the second-best predictor for progressive precocious puberty after the peak LH level. Furthermore, when we tested a combination of stimulated LH > 5.9 mIU/mL and US breast volume > 3.4 cc over stimulated LH > 5.9 alone, diagnostic accuracy increased confirming that ultrasonographic breast volume has an added value in predicting progressive precocious puberty.

We are aware of only two previous studies that investigated US for breast evaluation of girls with precocious puberty. Youn et al. [8] demonstrated in 90 girls (30 precocious puberty) that US staging of breasts positively correlated with bud diameter, age, bone age, and levels of pubertal hormones, but not with the difference between bone age and chronological age. In our study, we found significant correlation of ultrasound staging with bone age/chronological age, in addition to the other parameters. The most likely reason for this is the bone age/chronological age ratio being a more sensitive parameter for bone age advancement than the difference between bone age and chronological age. Alternatively, the higher number of subjects in our study could have led to significant correlation. In another study of 60 girls with early breast development, breast volume but not morphology was found to be a discriminative parameter for diagnosis of rapidly progressive precocious puberty [9] which is in line with our finding of breast volume > 3.4 cc having the highest OR and sensitivity and specificity after peak LH in predicting progressive precocious puberty.

In 52 obese children (7–12 years) with normal puberty, Yüce and Sevinç [10] demonstrated that the correlation between clinical (Tanner) staging and US staging of the breasts was poor (*r* = 0.19). US breast staging correlated better with estradiol levels, uterus long diameter, and ovary sizes than Tanner staging in obese girls in that study. In our unselected cohort, we found a correlation of *r* = 0.6 between Tanner staging and US staging of the breasts in 125 subjects. However, body mass index SDS was significantly higher in the 25 lipomastia patients compared to 100 physical examination positive patients and tended to be higher in group 2 than group 1, suggesting that in overweight girls physical examination alone can result in false diagnosis of thelarche. On the other hand, body mass index SDS was significantly higher in group 4 compared to group 1 suggesting that in some overweight girls, real glandular breast development can be masked under the fat tissue and missed by physical examination and better judged by US. In line with this, in subjects with body mass index > 1 SDS, correlation between Tanner stage and US breast volume was slightly weaker compared to those with body mass index SDS < 1. Notably, in one patient in group 4 who required treatment was an obese girl, her breast tissue was masked under the fat tissue and hence mistakenly judged as lipomastia on physical examination.

Elastography enables quantitative assessments of tissue stiffness [27]. Currently, two techniques are available for clinical use, which are strain elastography and shear-wave elastography. Shear-wave elastography has the advantage of delivering objective measurements and has been proven to be reproducible [28]. Shear-wave elastography is primarily used to characterize breast lesions to aid in reducing unnecessary biopsies in adults [29] However, premature breast development is a diffuse glandular process. Fibroglandular tissue has been shown to have higher stiffness compared to fatty tissue [30]. In this study, and (as far as we know) for the first time, we report results of shear-wave breast elastography in girls with precocious breast development. Glandular stiffness was higher in the progressive precocious puberty group which required treatment compared to the non-progressive group which did not require treatment. This indicates that elastography can assess hormonal effects on fibroglandular architecture but with low sensitivity and specificity (72.1 and 62.9 respectively). Recently, Keceli and Akyurek studied strain elastography in this context [31]. They also report a low differentiating power of elastography in girls with central precocious puberty and premature thelarche (sensitivity 71% vs 72.09% and specificity 61% vs 62.96%) [31]. Together with our findings it appears that although they have some value, neither shear-wave nor strain elastography have enough robustness to be used as a diagnostic method for this purpose.

A limitation of the study is that although highly competent and experienced in pediatric ultrasonography, only a single radiologist performed breast US. Confirmation of the findings by a second radiologist might have increased the robustness of our results. Another limitation of the study is the lack of elastography measurements in the subjects with no glandular breast tissue on US and the lipomastia group. This may have decreased the statistical power regarding the usefulness of elastography in the evaluation of early breast development.

## Conclusion

In a large group of girls referred for early breast development, we have demonstrated that US assessment of breast morphology, measurement of total breast volume, and elastography are helpful and non-invasive complementary tools in the assessment of breast maturation. Among these measures, total breast volume appeared to be the second-best independent risk factor, after the peak LH level for identifying girls with progressive precocious puberty. In multivariate analyses, elastography did not add value to standard B-mode US in predicting progressive precocious puberty.

## Data Availability

All data generated or analyzed during this study are included in this article. Further enquiries can be directed to the corresponding author.
